# Can CSF spectrophotometry for “Xanthochromia” be used to detect leaking subarachnoid aneurysms in patients with sickle cell anemia with negative MRI or CT angiogram despite hyperbilirubinemia?

**DOI:** 10.1002/ccr3.2775

**Published:** 2020-03-12

**Authors:** Wan Yung Siu, William Thomas, Rikin Trivedi, Alexandra Hogan, Umbareen Siddiqi, Anita Sarker, Martin Wolfgang Besser

**Affiliations:** ^1^ Department of Haematology Addenbrooke's Hospital Cambridge UK; ^2^ Department of Neurosurgery Addenbrooke's Hospital Cambridge UK; ^3^ Department of Anaesthetics Addenbrooke's Hospital Cambridge UK; ^4^ Cognitive Neuroscience & Psychiatry UCL Great Ormond Street Institute of Child Health London UK; ^5^ Department of Biochemistry Addenbrooke's Hospital Cambridge UK

**Keywords:** CSF xanthochromia, polycystic kidney disease, Sickle cell anemia, spectroscopy, subarachnoid hemorrhage

## Abstract

CSF (Cerebrospinal Fluid) xanthochromia by spectroscopy should not be dismissed in the context of hyperbilirubinemia in a patient with sickle cell anemia. Xanthochromia detected by spectrophotometry offers a vital clue that further invasive diagnosis is required.

## Graphical abstract

CSF xanthochromia by spectroscopy should not be dismissed in the context of hyperbilirubinemia in a patient with sickle cell anemia. Xanthochromia detected by spectrophotometry offers a vital clue that further invasive diagnosis is required.

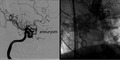

## 1 INTRODUCTION

A 17‐year‐old Afro‐Caribbean woman with known HbSS sickle cell anemia presented to the emergency department with a 5‐day history of thunderclap headache, neck stiffness, photophobia, nausea, and vomiting. She was afebrile, without focal neurological symptoms. In another hospital 3 days earlier, she was prescribed intravenous fluid and antibiotics and admitted overnight, but her symptoms persisted.

The initial presentation with acute headache, neck stiffness, and photophobia in this patient raised concern over meningism. The nausea and vomiting were in line with meningeal irritation or increased intracranial pressure. The clinical picture of a sudden onset severe headache appeared to be most consistent with subarachnoid hemorrhage in the absence of fever, though meningitis remained a possibility.

The patient organ system review was negative. She reported no recent trauma or increased cold exposure. She had mild sickle phenotype with intermittent joint pain which was well‐managed with ibuprofen. The headache was unlike her usual sickling pain. She had one previous admission for chest crisis precipitated by a bike accident. She had two transfusions in life; however, this had caused a persistent Anti‐LeA antibody. Her medications consisted of only ibuprofen, phenoxymethylpenicillin, folic acid, and cholecalciferol. Her father and the patient herself were known to have polycystic kidney disease, and she had several small cysts of the right kidney of up to 31 mm and was heterozygous for the PKD2 gene (c.1095‐2A > G) mutation. On examination, she was alert and oriented with a Glasgow Coma Scale of 15. She was apyrexial and hemodynamically stable with 100% oxygen saturation on 2L oxygen. Neurological examination revealed neck stiffness but was otherwise normal.

The most likely diagnosis that needs to be excluded is aneurysmal subarachnoid hemorrhage given her background of sickle cell anemia and history of polycystic kidney disease. Nevertheless, her symptoms could also represent a sickle crisis. A lumbar puncture was performed.

**Figure 1:**
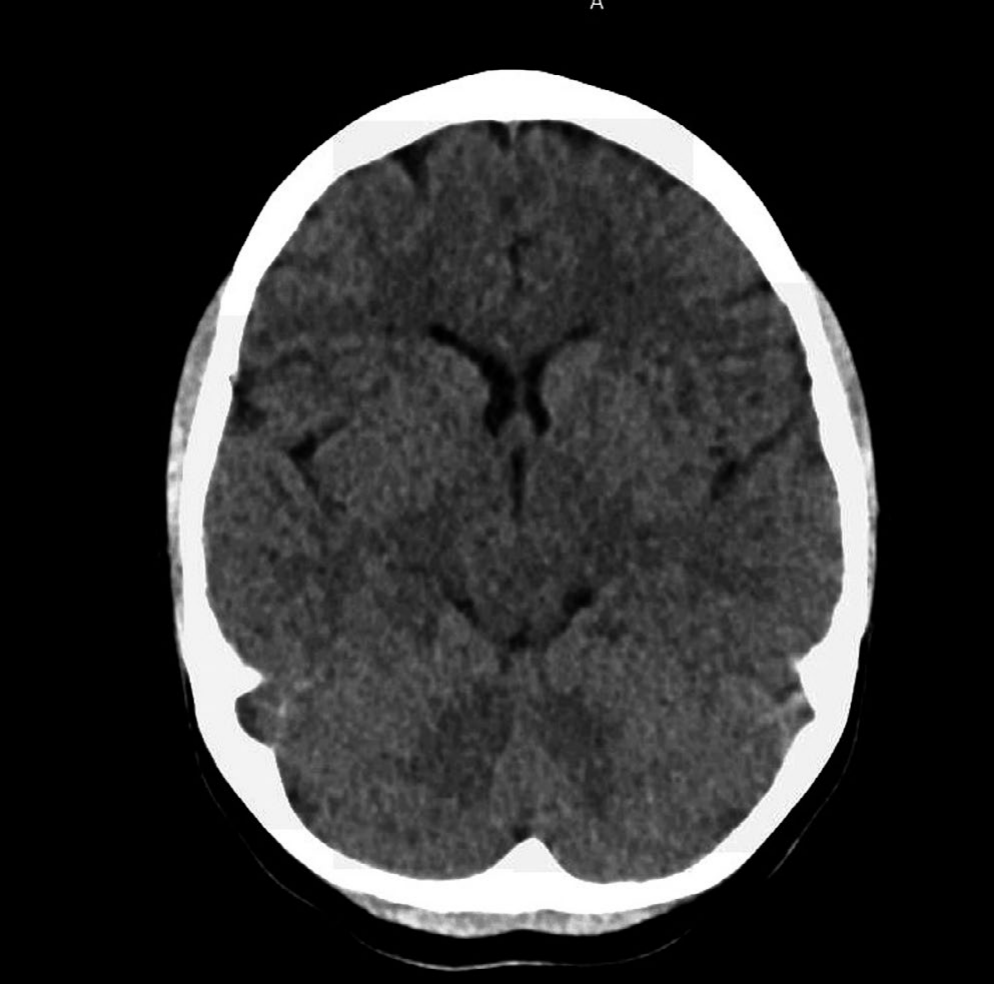
CT head without contrast performed at the time of presentation

**Figure 2:**
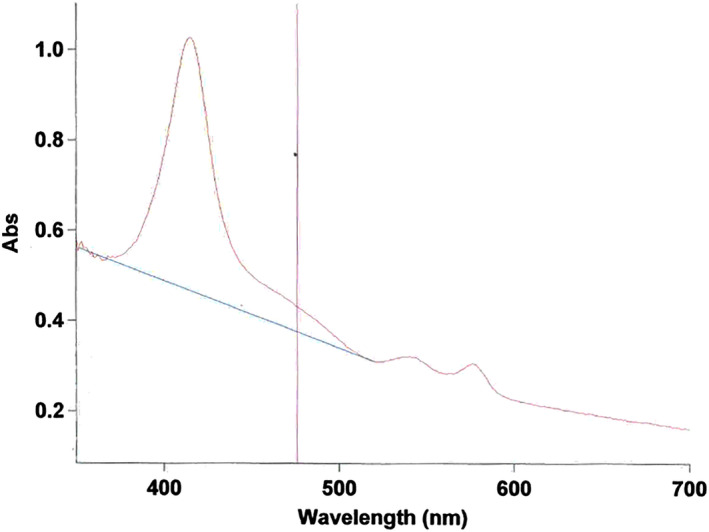
Spectrophotometric scan—Net bilirubin absorbance (NBA) is measured at 476 nm above a tangential base line

The patient's hemoglobin level was 70 g/L (comparable to her preadmission levels) with a mean corpuscular volume of 107.8 fL. The white cell count was 13.3 × 10^9^/L, with 9.19 × 10^9^/L neutrophils. The platelet count was 548 × 10^9^/L. The reticulocyte count was 247 × 10^9^/L. The total serum bilirubin was 100 µmol/L, serum total protein was 91 g/L, lactate dehydrogenase was elevated at 572 µmol/L. The C‐reactive protein level was <4 mg/L. The prothrombin time was 15.6 seconds and activated partial thrombin time was 24.8 seconds. The other liver function tests were normal. Blood film demonstrated polychromasia with Howell‐Jolly bodies and target cells, numerous sickle and boat cells. A noncontrast CT head was normal (Figure 1). The subjective macroscopic appearance of the CSF (Cerebrospinal Fluid) on visual inspection when viewed after centrifugation it appeared to be bloodstained. Microscopy of the CSF indicated 14 × 10^6^/L lymphocytes, 0 × 10^6^/L polymorph, 1796 × 10^6^/L red blood cells, 0.3 g/L protein, 2.7 mmol/L glucose (plasma glucose 5.1 mmol/L), and 1.5 mmol/L lactate. The spectrometry of cerebrospinal fluid showed 0.057 Ab units of net bilirubin absorbance, and 0.56 Ab units of net oxyhemoglobin. (CSF spectrophotometry scan in Figure 2).

This patient's macrocytic anemia was in keeping with chronic compensated hemolytic anemia with reticulocytosis. It was supported by other hemolysis markers, including hyperbilirubinemia and elevated lactate dehydrogenase, and the findings of peripheral blood smear with polychromasia, Howell‐Jolly bodies, target cells in the presence of sickle and boat cells. The leucocytosis with neutrophilia and with thrombocytosis are consistent with sickle cell anemia. The isolated prolonged prothrombin time is nonspecific and may be associated with vitamin K deficiency and phenoxymethylpenicillin in this patient. The absence of intracranial pathology on the noncontrast CT scan does not exclude the possibility of subarachnoid hemorrhage with a delayed presentation, as the sensitivity of the scan progressively declines with time. The analysis of cerebrospinal fluid could not rule out viral meningitis in the presence of lymphocytes, normal glucose, and protein, but bacterial meningitis is unlikely. Red cells in the CSF are considered to have a near 100% sensitivity for a subarachnoid hemorrhage but are less specific due to the possibility of a traumatic tap.

Notably, the spectrometry revealed an increased CSF bilirubin, which raises the possibility of subarachnoid hemorrhage, or hyperbilirubinemia from hemolysis in patient with sickle cell anemia.^1^, ^2^ A CT cerebral angiogram as a noninvasive test was requested to investigate a possible cerebral aneurysm.^3^

The patient was presumed to have subarachnoid hemorrhage and was prescribed nimodipine, vitamin K for prolonged prothrombin time and oxycodone. Ibuprofen was discontinued. The CT cerebral angiogram showed tortuous intracranial carotid and vertebral arteries, but no aneurysm. A digital subtracted angiogram was requested. The blood test on the second day of admission showed a fall in hemoglobin level to 55 g/L (2 g/L drop from baseline of 70 g/L), and hemoglobin electrophoresis revealed 3.8% HbA2, 7.6% HbF and 88.6% HbS. Two units of red cells were transfused. Two further units of red cells were given to lower the preangiogram HbS percentage to 43.6%.

The finding of the CT cerebral angiogram suggests a possibly early sign of sickle cell vasculopathy involving the intracranial carotid and vertebral arteries, but the scan was otherwise negative. The lack of evidence for subarachnoid hemorrhage and aneurysm on both the noncontrast CT scan and the CT cerebral angiogram favors the hypothesis of painful sickle crisis instead of aneurysmal subarachnoid hemorrhage. However, in view of the patient's history with persistent thunderclap headache and a raised CSF bilirubin in cerebrospinal fluid, an underlying cerebral aneurysm has to be excluded by invasive angiography. With regards to the high HbS level in this patient, preangiogram we transfused the patient to a HbS level below 50% to reduce the risk of sickle crisis with radiological contrast media. Bacterial or viral meningitis was ruled out in the meantime by CSF culture and PCR.

**Figure 3:**
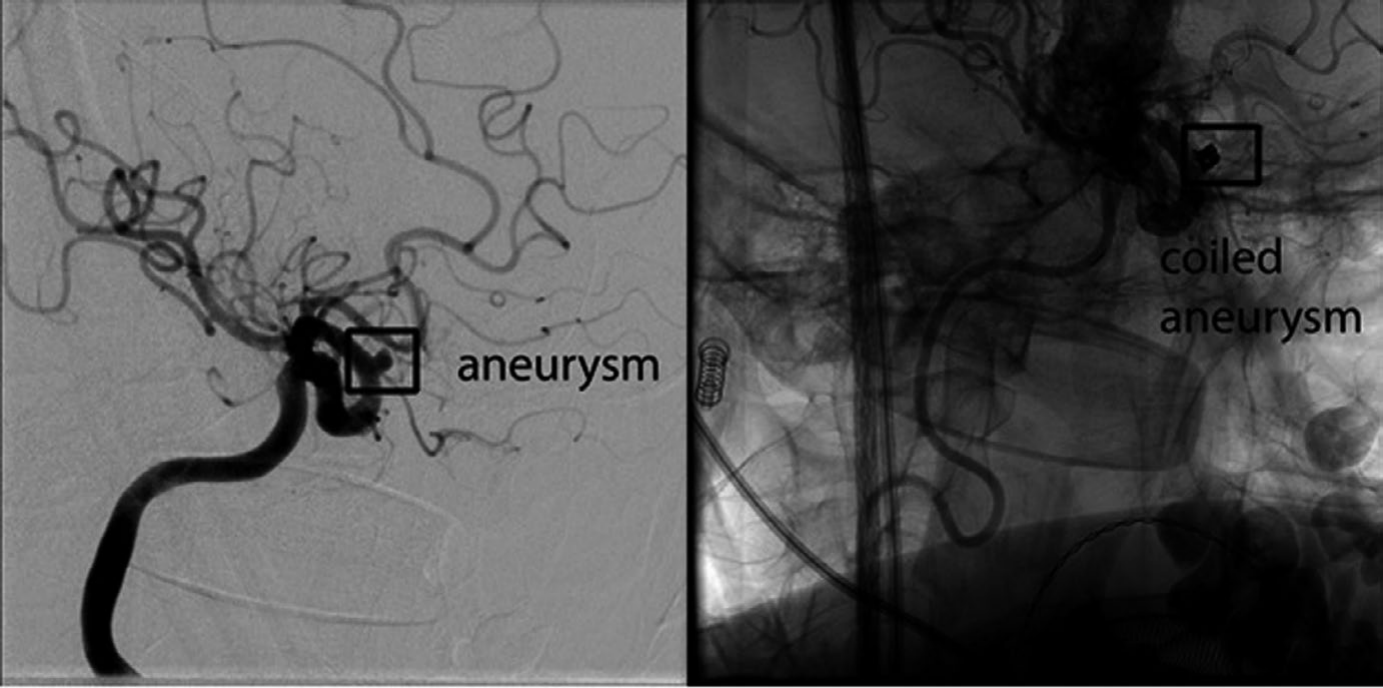
Right carotid angiogram shows a wide‐neck aneurysm projecting superiorly and medially from right internal carotid artery at the level of ophthalmic artery. Second image shows the angiogram after successful 3.5 × 18 mm braided stent placement followed by 3 coils

The cerebral angiogram revealed a 3 mm wide‐neck aneurysm projecting superiorly and medially from the right internal carotid artery at the level of ophthalmic artery (Figure 3). In view of the patient's history and increased CSF bilirubin in cerebrospinal fluid, endovascular coil embolization was planned. Preoperatively, her hemoglobin level was 103 g/L with 39.1% HbS. She then had venesection of 300 mL and was transfused one unit of packed red cells. Subsequent hemoglobin level was 109 g/L, and HbS was 32.2%. The preoperative target HbS% was based on published cases from the literature.^4^ The purpose for venesection and transfusion, with the aim of the hemoglobin level at 100‐110 g/L and HbS at 30%, is to reduce the risk of peri‐operative complications.

The patient underwent uneventful stent coiling of the right carotid‐ophthalmic aneurysm. She reported a resolution of headache and other symptoms postoperatively. She was discharged with long‐term aspirin and a 3‐month course of clopidogrel and remains well.

## 2 DISCUSSION

This patient presented with classical clinical symptoms of subarachnoid hemorrhage, but the diagnostic difficulties lie in the interpretation of xanthochromia with negative neuroimaging. It is difficult to distinguish a traumatic tap from a genuine subarachnoid hemorrhage. Spectrophotometric measurement of CSF bilirubin can be very helpful in distinguishing these 2 possibilities.^5^

After a subarachnoid hemorrhage, red blood cells are released and cell lysis occurs so oxyhemoglobin will be present within a few hours. Oxyhemoglobin split into hem and globin, hem is then metabolized to biliverdin by an inducible hemoxygenase followed by a reduction to bilirubin by biliverdin reductase. Both enzymes are present in the cells of the CNS. However, oxyhemoglobin is also released by the in vitro lysis of red blood cells and it is only bilirubin that is formed slowly by in vivo conversion and is therefore a more specific marker for subarachnoid hemorrhage.

The term CSF xanthochromia refers to a yellow discoloration of CSF associated with the presence of hemoglobin breakdown products such as bilirubin. Spectrophotometry is the measurement of the intensity of light at selected wavelengths and depends on the light absorbing properties of the substance being analyzed. Spectrophotometry of CSF in the visible region is, therefore, considered more sensitive and objective than visual examination, with peaks at 415 and; 440‐460 nm indicating the presence of hemoglobin (Hb) and bilirubin, respectively.

**Figure 4:**
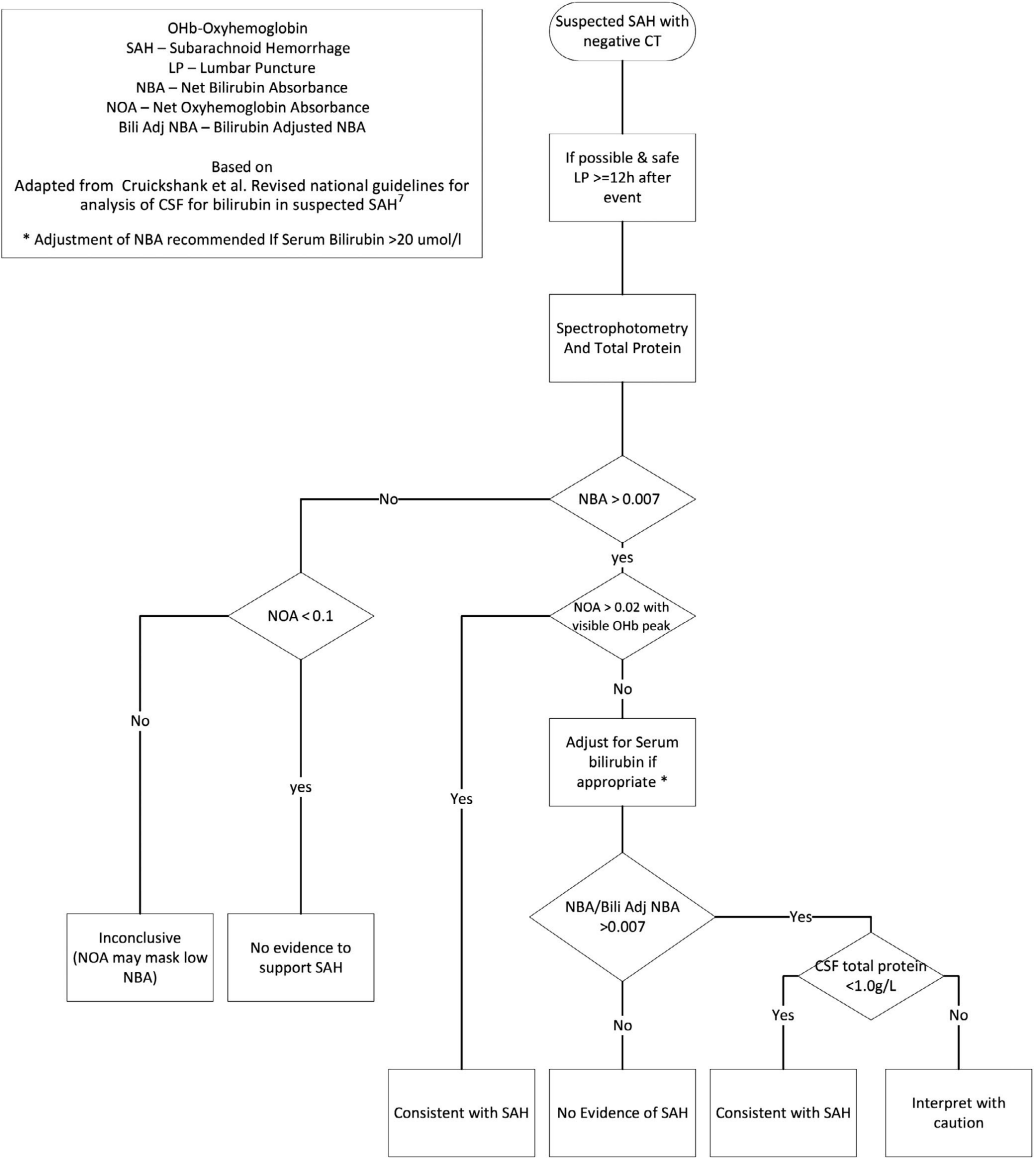
Flow Diagram how to interpret CSF Xanthochromia results adapted from Cruickshank et al^7^

After collection of the CSF, the last bottle collected undergoes centrifugation. The supernatant is then scanned between the wavelengths of 350 and 600 nm using a recording spectrophotometer. The net bilirubin absorbance (NBA) is measured at a wavelength of 476 nm and the net absorption of any oxyhemoglobin peak is also measured (NOA). It is a manual rather than an automated procedure and can be quite labor intensive requiring inspection of the scan for interpretation. In the UK an algorithm is used to aid interpretation (see Figure 4).

However, in patients with sickle cell anemia, the high level of serum bilirubin from chronic hemolysis may raise concern over a false positive finding of elevated CSF bilirubin in cerebrospinal fluid. Although CSF bilirubin is regarded as the primary criterion in diagnosing subarachnoid hemorrhage in patients with negative CT scans,^6^ it is known that hyperbilirubinemia with serum bilirubin concentration >20 µmol/L may give rise to protein‐bound bilirubin in cerebrospinal fluid in the absence of intracranial bleeding.^1^, ^7^ In such clinical scenarios, a formula is proposed to aid the diagnosis of subarachnoid hemorrhage when a patient has a net bilirubin absorbance >0.007 and serum bilirubin >20 µmol/L^7^:

$$\begin{array}{cc} \text{Predicted}\;\text{Absorbance}= & \frac{\text{CSF}\;\text{total}\;\text{protein}\;(g/L)}{\text{Serum}\;\text{total}\;\text{protein}\;(g/L)}\\ \; & \times \;\text{Serum bilirubin}\;\mu \text{mol}/L\times 0.042\;\text{AU} \end{array}$$

Then, AdjustedNetBilirubinAbsorbance=MeasuredNetBilirubinAbsorbance-PredictedAbsorbance

In this patient, the adjusted net bilirubin absorbance is 0.043 Ab units, which is greater than the cutoff of 0.007 Ab units suggesting that the CSF bilirubin would not be totally accounted for by the increase in serum bilirubin. A further comparison can be made from the cerebrospinal fluid analysis in her at a subsequent admission for headache, fever, vomiting, and back pain 3 months later when she underwent repeat lumbar puncture due to concerns about a new subarachnoid hemorrhage. This time although the net bilirubin was 0.012 Ab units, the serum bilirubin was 172 μmol/L, and the calculation of the adjusted net bilirubin using the formula above indicated that the increased CSF bilirubin was probably totally accounted for by increase in serum bilirubin and therefore not supportive of subarachnoid hemorrhage. It shows the significance of accurate diagnosis and high index of suspicion should remain if patients with sickle cell anemia are found to have xanthochromia and the importance of paired measurements of serum bilirubin and total protein to enable the calculation of adjusted bilirubin.

The negative neuroimaging prompted a cerebral angiogram to confirm the diagnosis and further endovascular occlusion of the aneurysm in this patient. Evidence from the literature suggested that radiological contrast, general anesthesia, and surgery may precipitate sickling and vaso‐occlusion by hypoxia, acidosis, and hypothermia.^8^, ^9^, ^10^, ^11^, ^12^, ^13^ Recommendations including preangiogram and preoperative transfusion, adequate hydration, use of contrast media with low‐osmolality or nonionic contrast are put forth to reduce the peri‐procedural risk.^10^, ^11^, ^13^, ^14^, ^15^, ^16^Previously top‐up or exchange transfusion, with a target of HbS, ranging from <30% to <50%^10^, ^15^, ^16^, ^17^, ^18^, ^19^, ^20^ and a hemoglobin of 100 g/L as the optimum range for cerebral blood flow^10^, ^17^, ^18^, ^19^ In someone with preformed red cell antibodies who has a lifelong increased need for red cell transfusion, the risk of further antibody formation, however, has to be balanced against the benefits of reducing chronic arterial intimal hyperplasia that was shown in the studies.^9^, ^10^ This is in keeping with the benefits demonstrated in the randomized, controlled TAPS trial comparing no preoperative transfusion (top‐up simple transfusion if hemoglobin level <90 g/L) or partial exchange transfusion if hemoglobin level ≥90 g/L targeting 100 g/L and HbS <60% in patient with sickle cell disease supported that preoperative transfusion was associated with a decrease in peri‐operative complications for low‐ and medium‐risk surgeries.^4^

Autosomal dominant polycystic kidney disease is associated with an increased risk of cerebral aneurysms affecting up to 7% of patients. This increased at a risk of 15% if there are first degree relatives with subarachnoid hemorrhages or aneurysms.^21^, ^22^ Screening of asymptomatic is recommended by MRI angiogram or CT but only in high risk cases.^21^, ^22^

Sickle cell anemia in its own right predisposes to vascular abnormalities that can include aneurysm of the cerebral arteries. In a recent case series, over 15 years 7/26 patients with sickle cell disease also had aneurysm originating from the Caroto‐ophthalmic artery.^23^ This location is uncommon for polycystic kidney disease making a sickle etiology in our patient more likely.^17^, ^24^, ^25^

This case demonstrates the clinical challenges in diagnosing and managing aneurysmal subarachnoid hemorrhage in patient with sickle cell anemia. A high level of clinical suspicion with appropriate investigations led to the definitive diagnosis and correct treatment and shows that the clinical utility of CSF spectrophotometry is maintained in sickle cell anemia and that positive findings need to be investigated as in any other patient.

## CONFLICT OF INTEREST

MWB states the following conflicts of interest: Advisory boards: Novartis, Pharmacosmos, Haemosonic, Werfen. Study support: Novartis, Haemosonics and Pharmacosmos. Speakers honoraria: Stago. All other authors do not state any relevant conflicts of interest.

## AUTHOR CONTRIBUTIONS

WYS prepared the manuscript and conducted initial literature search, WT prepared manuscript, RT prepared manuscript, AH prepared manuscript, US prepared manuscript, AS prepare manuscript, MWB critically reviewed manuscript, and coordinated author input for final manuscript submission.
